# Exploring the Nexus Between E-Business Processes and Organizational Performance: Can Technological Opportunism Play Any Role?

**DOI:** 10.3389/fpsyg.2022.896527

**Published:** 2022-05-30

**Authors:** Mohammad Ahmad Al-Omari, Mahmoud Radwan Hussein AlZgool, Umair Ahmed, Munwar Hussain Pahi, Qais AlMaamary

**Affiliations:** ^1^College of Business, Al-Ain University, Al Ain, United Arab Emirates; ^2^College of Administrative and Financial Sciences, Gulf University, Sanad, Bahrain; ^3^Department of Business Studies, Arab Open University, A’ali, Bahrain; ^4^PAF KIET, Karachi, Pakistan; ^5^School of Business Management, Universiti Utara Malaysia, Kedah, Malaysia

**Keywords:** online procurement, online channel management, online service delivery, organizational performance, manufacturing businesses

## Abstract

The digitization of business processes has gained much scholarly and practical attention in the recent past. To understand their effectiveness, particularly in connection to organizational performance, the current study developed and tested a comprehensive framework. Through obtaining data from 350 manufacturing businesses, the study investigated using Smart PLS3 and found a significant influence of online procurement, channel management, and service delivery capabilities on organizational performance. Furthermore, the study also found the significant direct and moderating potential of technological opportunism to harness organizational performance. The study concludes that e-business processes serve as crucial resources and capabilities for businesses to achieve their goals and objectives. The study offers contributory implications for theory and practice indicating for strategic investment of e-business processes for businesses aiming to boost their performance.

## Introduction

The Internet and technological revolution have set a trend of investment in the transformation of conventional businesses processes, thus enhancing functioning and collaboration (Zhao et al., 2008; Zhu et al., 2020). Scholarly evidence suggests that e-business has progressed to be one of the pivotal management practices in the competitive world (Melão, 2009). Typically, e-business processes enable organizations to manage and organize their resources effectively and efficiently (e.g., Soto-Acosta and Meroño-Cerdan, 2008). Following the explanations of Zhu et al. (2015), we operationalize e-business processes as an approach to business that operates through the Internet-enabled flow of information across the organization and external parties of interest to enhance the capitalization of digital activities. While the transformation of conventional businesses to digital is consistently showing an upward trend (e.g., Fahey et al., 2001), it has received even more attention due to the ongoing COVID-19 Pandemic (Bhatti et al., 2020). Nonetheless, despite the massive implementation and application of e-business processes, firms continue facing challenges in obtaining optimum value from their e-business processes (Zhu et al., 2020). To authors, this indicates a need for further attention and investigation to outline how and in what specific business processes an organization needs to work to achieve the desired end outcomes.

Typically, the end objective of every organization is performance, and recent studies have underlined the acute role of IT-enabled businesses processes in enhancing business performance (Rai et al., 2006; Wu et al., 2011; Olusegun et al., 2020). Yet still, the role of IT-enabled business process limited in objective grounds is not highlighted. For example, little is known about utilizing IT-enabled services to deal with external entities, such as dealing with suppliers on procurement matters online, managing products online (channel management), and providing online service to customers (Zhu et al., 2020). This is because most studies have focused on investigating the significance of inter-firm e-business processes (Shi and Liao, 2015; Chi et al., 2017; Tsironis, 2021). Hence, there is limited scholarly knowledge on the significance of e-business processes catering to external parties to help boost business performance. Typically, literature on process components suggests three main features of e-business that cater to digital operations of the business for pursuing transaction, service, and collaboration. Some scholars refer to these as e-business process capabilities that include online procurement, online channel management, and online service delivery (Voola et al., 2012a). These three e-business process capabilities can be referred to as IT-enabled abilities and operations of the business to facilitate information sharing, conduct and coordinate supply chain activities, and provide service to relevant stakeholders digitally (Johnson and Whang, 2002).

The technological capability can be seen as a strategically vital feature facilitating an organization to achieve a competitive advantage (Tsai, 2004; Tang et al., 2020). Accordingly, for the effective capitalization of IT-enabled processes, the business must be proactive in sensing and responding to technological developments in their respective sector for better performance (Voola et al., 2012b). This could be termed even more crucial for businesses with e-processes to adopt new technologies in a timely manner for long-term competitive advantage and performance (Voola et al., 2012a; Chen and Lien, 2013). Hence, we assert that technologically opportunistic firms will be actively scanning for developments and deploying necessary actions for their implementation to compete better and boost the capitalization of e-business operation processes for even better performance. However, despite its acute importance, less is known about technological opportunism, particularly its moderation role (e.g., Voola et al., 2012b). Moreover, pertaining to components related to e-business operations capabilities such as online procurement, online channel management, and online service delivery and their association with business performance, and the interaction effect of technological opportunism is yet to be tested. Conclusively, the above discussions triggered two important questions to answer:

RQ1: Can the three e-business operation capabilities enhance business performance?

RQ2: Can technological opportunism moderate the association between the three e-business operation capabilities and business performance?

In the study, we investigate the three major e-business processes (i.e., procurement, channel management, and service delivery) to see how they relate to enhancing organizational performance in the manufacturing sector, following the moderation of technological opportunism. The current study uses resource orchestration theory (Sirmon et al., 2007, 2011) as a foundation to explain and investigate the nexus between the aforementioned variables. Resource orchestration theory is an extension of the resource-based perspective which allows viewing how leveraging capabilities can enhance performance prospects. Prominent studies have used resource orchestration theory as the theoretical lens to examine the impact of different resources and organizational capabilities on end results (Du et al., 2018; Burin et al., 2020). The current study, looking at the three e-components as notable resources for business (Zhu et al., 2020), attempts to enhance the knowledge on the significance of online procurement capabilities, online channel management, and online service delivery toward boosting organizational performance following the moderation of technological opportunism; a framework that remained overshadowed in the extant literature.

The study brings four notable contributions starting with unveiling the role and importance of e-business processes, particularly in the manufacturing sector. Accordingly, the study extends previous studies (Rai and Tang, 2010; Li et al., 2020) by outlining how the three important e-business processes instrumentally help enhance end performance prospects, thus outlining the need to better structure and strengthen such e-resources. Furthermore, the study underlines how a proactive outlook toward technological developments and their capitalization can be valuable to harness the use of e-business capabilities for maximum organizational performance. Lastly, the study results will also provide insights for practitioners to strengthen e-business capabilities for better performance strategically.

The remainder of the paper is structured as follows: Section 2 presents a critical appraisal of the literature and hypotheses. Section 3 discusses methodology, and Section 4 talks about analysis and interpretation. Section 5 provides a detailed discussion on the results, followed by Section 6, shading light on implications for theory and practice and scope for future studies on the topic.

## Literature Review and Hypotheses Development

### E-Business Processes and Organizational Performance

Following the assertions of Chang et al. (2013), online procurement capability denotes to the digital capacity of a business to perform procurement activities such as product realization, service identification, negotiation, transaction processing, product coordination, and material management and demand planning. Accordingly, Wu et al. (2007) defines e-procurement as the utilization of IT-enabled processes to conduct business transactions related to materials, production scheduling, and services. The second definition connects more with the understanding current study present regarding the concept of e-procurement capability. Typically, responsive e-procurement capability is seen as one of the important entities for businesses aspiring to boost end results. For example, studies have outlined the effect of e-procurement technologies in harnessing procurement performance (Quesada et al., 2010). Accordingly, e-procurement is also found to improve the business’s efficiency and IT capabilities, leading toward boosting business performance (Chang and Wong, 2010). Studies also suggest that e-procurement has revolutionized the integration of businesses with suppliers, production partners, and service providers (Madzimure et al., 2020). E-procurement capability can improve day-to-day business activities related to material management and production scheduling, thus translating to improved company performance (Sánchez-Rodríguez et al., 2019). Hence, the authors speculate that the business’s e-procurement capabilities will help businesses effectively negotiate procurement, share and coordinate schedules with suppliers, and support online material management and demand management. Therefore, we posit as:

*H1*: There will be a positive association between online procurement capability and organizational performance.

Online channel management capability refers to a business’s ability to perform unified online promotion, product launches, pricing, e-transactions, and order fulfillment (Oh et al., 2012). Online channel management deals with transaction processes to support order management, sharing marketing policies and support for distribution and promotion, order catalog and online distribution to support pricing and product launches, and managing production schedules for online support and order fulfillment. Online channel management systems can instrumentally help engage the internal process and the distributor engagement to boost performance prospects (Zhu et al., 2020). It also plays a notable role in integrating resources from a wide range of distributors for maximum operational value for the business (Zhu et al., 2015). Thus, online channel management capability can serve as a foundation to develop a platform whereby the organization actively shares information with the distributors and help support pricing, promotion, and other transactional activities with the distributors, which would reduce the operational cost (Xia and Zhang, 2010). Accordingly, this may also help boost organizational performance, a link that has not received a fair amount of scholarly attention. Thus, we posit as:

*H2*: There will be a positive association between online channel management capability and organizational performance.

Online service delivery denotes an organization’s online capability to perform online services pertaining to customer communication, sales support (after-sales), demand tracking, and response to customer queries (Roberts and Grover, 2012). Online service delivery platforms enable businesses to integrate services to support customers and address their demands (Setia et al., 2013). Moreover, healthy online service delivery systems and platforms play an instrumental role in improving customer loyalty (Suhartanto et al., 2019), thereby improving an organization’s end results. Notably, the rise of the use of technology and the Internet in business has tremendously shifted the need for businesses for such prospects to improve their performance (e.g., Ahmed et al., 2016; Attaran and Woods, 2019; Martín-Peña et al., 2019; Ho et al., 2020). Thus, it can be asserted that providing various online services to support customer interaction, value-added features on the website to attract new customers, and after-sales and order support services followed by feedback and suggestion consideration can help boost operational effectiveness and efficiency, harnessing organizational performance. Thus, we posit as:

*H3*: There will be a positive relationship between online service delivery capability and organizational performance.

### Role of Technological Opportunism

The current study believes that technological opportunism will complement the associations of online procurement capability, channel management capability, and service delivery capability to further organizational performance. We define technological opportunism as an organizational process that facilitates the prompt identification and capitalization of appropriate technological developments (Srinivasan et al., 2002a).

Therefore, a firm with technological opportunism will be more proactive in enriching its online procurement, channel management, and service delivery capabilities to bring effectiveness and efficiency to business processes, which will boost organizational performance (Sarkees, 2011). Firms with higher technological opportunism will be better than those not actively responding to technological developments, potentially limiting their competitiveness and organizational performance. Thus, robust responsiveness toward technological developments would be resourceful to address procurement, channel management, and service delivery lapses (if any) while consistently improving them for better organizational performance. Importantly, recent studies have also supported the strategic vitality of technological opportunism (Orlandi et al., 2020) and how it can boost various performance-based prospects (Feiz et al., 2017; Urban and Maphumulo, 2021). Resultantly, these shreds of empirical evidence have confirmed the role and vitality of technological opportunism for businesses aspiring to boost performance in the world rapidly digitizing. In addition, the explanation of Resource Orchestration Theory also justifies the moderation of technological opportunism since information and technology capabilities can help organizations further there existing resources such as e-business processes in the case of current study to achieve optimal levels of performance (see further., Wales et al., 2013; Wang et al., 2020). Therefore, following the empirical assertions of Voola et al. (2012b), we propose that technological opportunism will help strengthen the direct association of online procurement, channel management, and service delivery capability to improve organizational performance. Sadly, no shreds of empirical evidence are available outlining the interaction effect of technological opportunism on these IT-enabled business processes toward harnessing business performance. Thus, we posit as:

*H4*: There will be a positive relationship between technological opportunism and organizational performance.

*H5*: Technological opportunism will moderate the relationship between online procurement capability and organizational performance.

*H6*: Technological opportunism will moderate the relationship between online channel management capability and organizational performance.

*H7*: Technological opportunism will moderate the relationship between online service delivery capability and organizational performance.

## Control Variables

In order to limit firm-based effects on the results, the current study controlled three variables, e-business duration, industry, and size. E-business duration refers to the number of years e-business processes were implemented. The study controlled this since such initiatives take time to show their impact on performance (Zhu et al., 2020). Likewise, we controlled the industry to intercept the performance variations based on industry characteristics (Rai and Tang, 2010). Accordingly, the implementation of e-business processes can increase the variable costs depending on the size of the business (Wu et al., 2003). Thus, it is essential to control its effects (Chandy and Tellis, 1998) to obtain objective results.

## Methodology

### Data Collection

The current study collected data using survey technique from manufacturing firms in United Arab Emirates that primarily interact with customers, suppliers, and distributors through using online business technologies. The details of these companies were obtained from the business directory portal for United Arab Emirates, which lists 7,820 operational entities. The researchers chose the UAE market due to its thriving business environment, whereby the country is striving to boost industrialization. Therein, we shortlisted 561 organizations operating in Dubai that detailed full-scale production-based businesses. From where we extracted industry stratified random sample of 350 organizations. Following the guidelines of Zhu et al. (2006), we used the main informant approach and approached one senior IS manager or operations manager who is most informed about the online systems and how they are utilized pertaining to online procurement, channel management, and service delivery through a screening question. The study also examined the consistency of the data and any existence of potential biases on the main variables. The study found that the size of the firms reflected a balance of small, medium, and large enterprises.

Through self-administered questionnaires, emails with invitations stating the purpose of the study to the targeted managers. The authors also attempted to contact concerned individuals through telephone (wherever possible) to obtain maximum response. The managers who agreed were emailed the questionnaire. The respondents were given a total of 3 weeks to respond, and follow-up emails were sent for gentle reminders on a weekly basis. The study received 288 responses out of which 19 were found to be inappropriately filled. Thus, 269 responses were taken further for analysis and interpretation. The final response rate was 76.8% which is satisfactory for survey research based on the guidelines of Sekaran and Bougie (2016).

### Measurements

The work of Mishra et al. (2007) and Soto-Acosta and Meroño-Cerdan (2008) was used to procurement capability. Accordingly, the four-item scale for channel management capability was utilized from Oh et al. (2012) and Eng (2008) was referred for online service delivery. Furthermore, the study adapted the scale by Srinivasan et al. (2002b) to investigate technological opportunism. Lastly, Hooley et al. (2005) was considered to examine organizational performance.

### Model Evaluation

**Figure 1:**
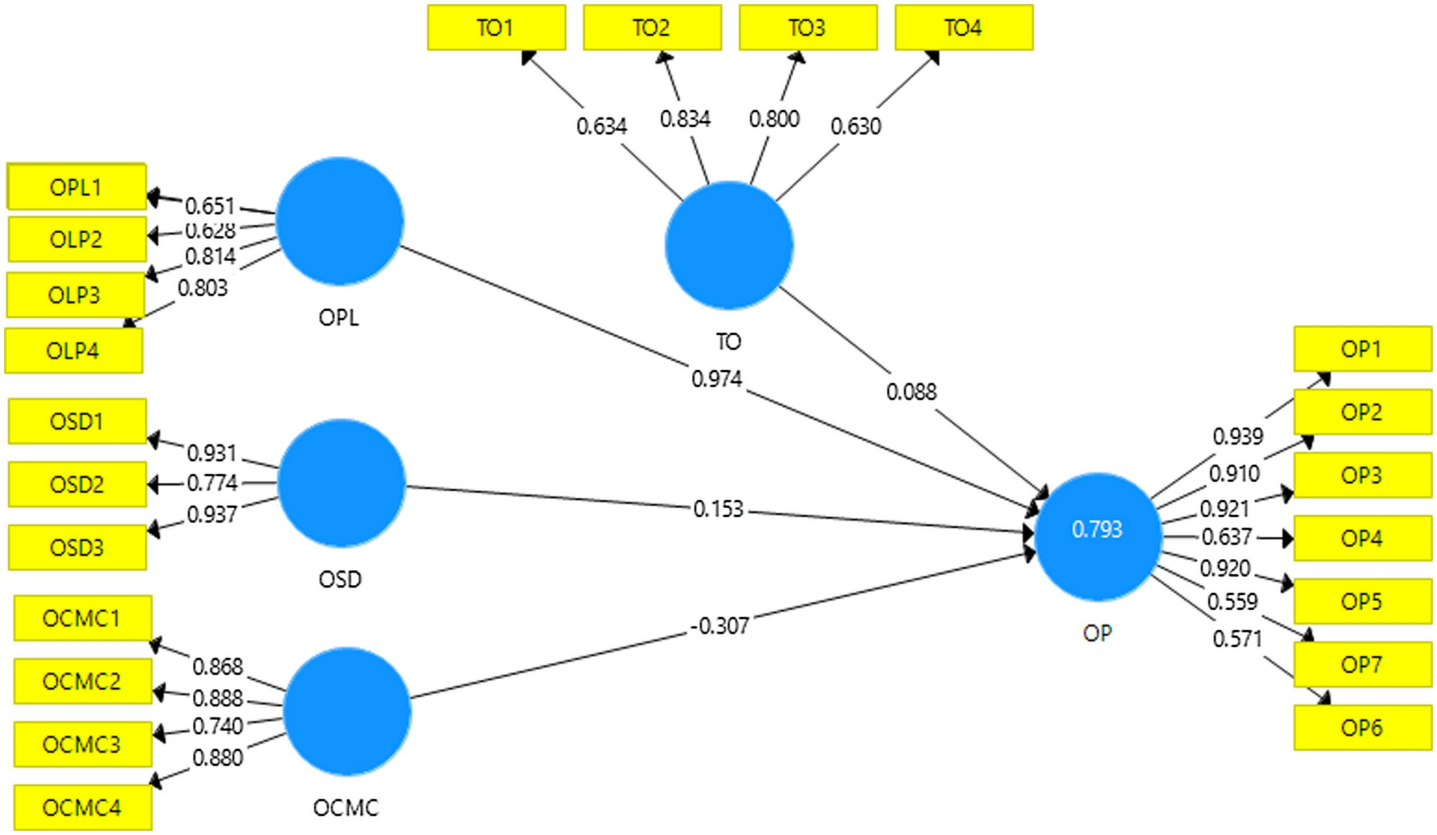
Measurement model.

**Figure 2:**
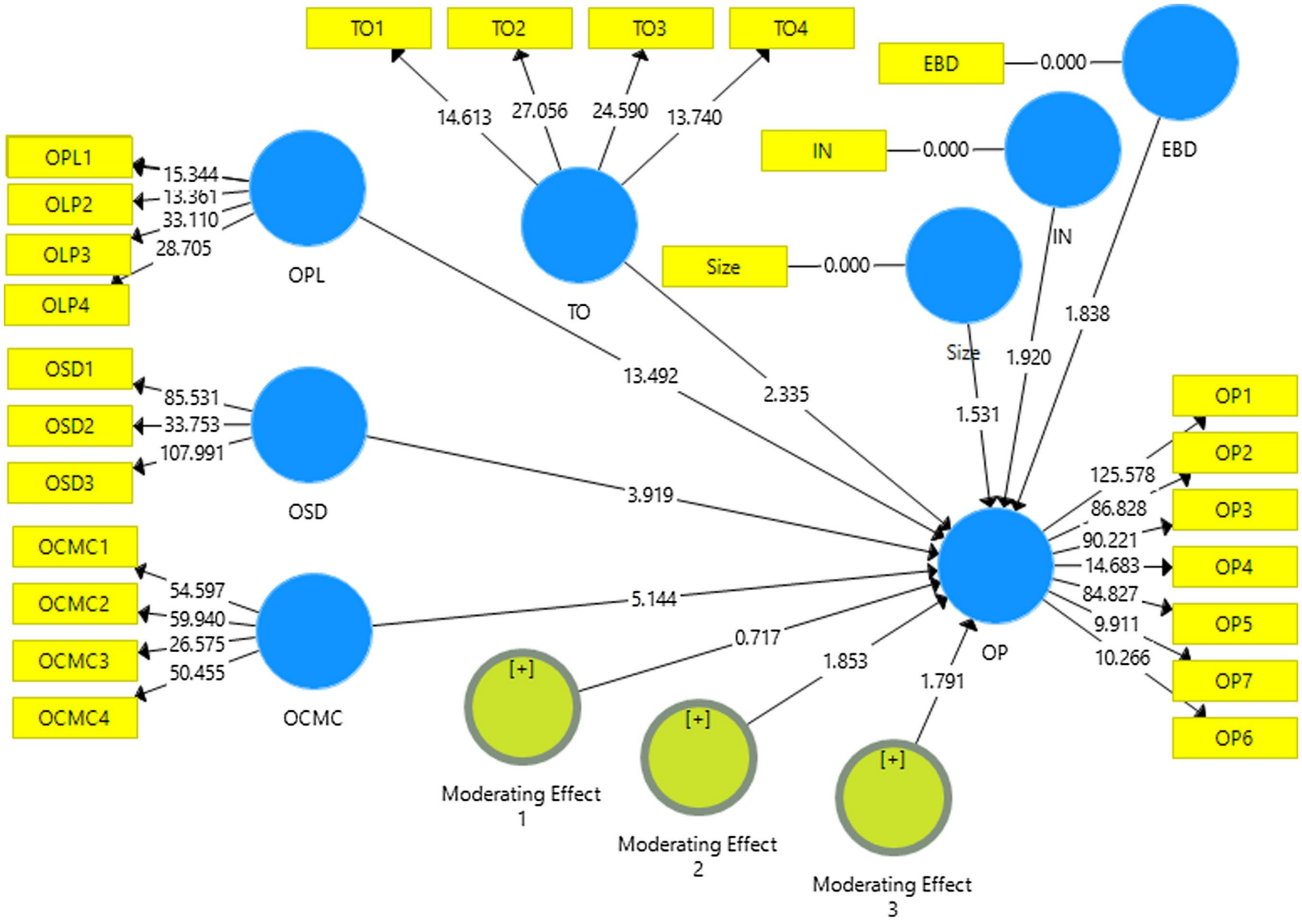
Structural model.

Structural equation modeling using Smart PLS 3.2 was used to assess the hypothesized relationships in the current study (Ringle et al., 2015). Smart PLS has received much scholarly appreciation from prominent studies due to its robust statistical analysis and interpretation features in the recent past (Umrani et al., 2020; Ahmed et al., 2021). The study followed the guidelines of Anderson and Gerbing (1988) and applied the two-stage process. At first, we tested the measurement model to examine the conceptual model’s psychometric properties followed by the structural model to assess the significance of the hypothesized relationships. Figure 1; Table 1 show the measurement model results regarding individual item loading, composite reliability, and average variance extracted. On the suggestion of Hair et al. (2017), all external loadings should be greater than 0.5. The measurement model results in Table 1; Figure 1 indicate, ranging between 0.562 and 0.920, thus meeting the recommended cut-off. The second requirement of the measurement model is assessing composite reliability. Based on the recommendations of Hair et al. (2017), it should be greater than 0.7. Composite reliability was determined, and the result of Table 1; Figure 2 indicates that CR scores for all constructs ranged between 0.818 and 0.909, thus meeting the required threshold. Finally, we assessed the average variance extracted. Following the suggestions of Hair et al. (2017), the AVE scores should be 0.5 or above. Results of the measurement model in Table 1; Figure 1 show that AVE scores ranged between 0.534 and 0.716. Taken together, the results confirmed that the constructs meet the requirement of the measurement model and are fit for further data analysis and interpretation.

**Table 1:** Measurement.

Constructs	Item	loading	AVE	CR	*R*-square
Online channel management capability			0.716	0.909	
	OCMC1	0.868			
	OCMC2	0.887			
	OCMC3	0.740			
	OCMC4	0.880			
Online procurement			0.549	0.858	
	OLP1	0.788			
	OLP2	0.651			
	OLP3	0.814			
	OLP4	0.803			
Organizational performance			0.635	0.921	0.790
	OP1	0.938			
	OP2	0.910			
	OP3	0.920			
	OP4	0.637			
	OP5	0.919			
	OP7	0.562			
	OP6	0.574			
Online service delivery			0.781	0.914	
	OSD1	0.931			
	OSD2	0.774			
	OSD3	0.937			
Technological opportunism			0.534	0.818	
	TO1	0.634			
	TO2	0.834			
	TO3	0.800			
	TO4	0.631			

Fourth, discriminatory validity was accessed on the recommendation of Fornell and Larcker (1981) that the square root of the AVE should be greater than the correlations among latent constructs. The results in Table 2 confirm discriminant validity.

**Table 2:** Discriminant Validity.

	1	2	3	4	5
OCMC	0.846				
OP	0.699	0.797			
OPL	0.735	0.670	0.746		
OSD	0.644	0.689	0.704	0.884	
TO	0.599	0.624	0.653	0.551	0.731

### Structural Model

The current study ran a bootstrapping procedure with 5,000 resamples following Hair et al.’s (2017) guidelines to examine the structural model to obtain bootstrap confidence intervals, *t* and *p* values. The study controlled the investigated model for any confounding effect of length of e-business duration, industry, and firm size on organizational performance. The results indicated that the duration of e-business has a significant negative controlling effect on organizational performance (*β* = –0.045; *p* = 1.838). Accordingly, the industry was also found to have a significant controlling effect on organizational performance (*β* = 0.229; *p* = 1.920). In contrast, firm size did not showcase any controlling effect on organizational performance (*β* = 0.184; *p* = 1.531).

In connection to the results for the hypotheses, Table 3; Figure 2 show that all direct hypotheses, H1, OPL Online procurement capability, H2, OCMC Online channel management capability hypothesis, and H3, OSD Online service delivery, were found to be statistically significant in relationship with organizational performance (*β* = 0.972, *t* = 13.492, *p* < 0.000), (*β* = 0.317, *t* = 5.144, *p* < 0.000), and (*β* = 0.159, *t* = 3.919, *p* < 0.000). Accordingly, the study also tested the association between technological opportunism and organizational performance and found it to be promising (*β* = 0.092; *t* = 2.335). Technological opportunism also moderated the relationship between online channel management and organizational performance (*β* = 0.134; *t* = 1.853). Furthermore, the study also found significant moderation of technological opportunism on the association between online service delivery capability and organizational performance (*β* = 0.131; *t* = 1.791). In contrast, technological opportunism did not significantly affect the online procurement capability and organizational performance association (*β* = −0.031; *t* = 0.717). Table 3; Figure 2 provide further details in this regard.

**Table 3:** Structural model.

Relationships	Beat	STD	T-statics	*P* values
OPL→ - > OP	0.972	0.072	13.492	0.000
OCMC→OP	0.317	0.062	5.144	0.000
OSD→OP	0.159	0.041	3.919	0.000
TO→OP	0.092	0.039	2.335	0.010
Moderating Effect 1→OP	−0.031	0.044	0.717	0.237
Moderating Effect 2→OP	0.134	0.072	1.853	0.032
Moderating Effect 3→OP	0.131	0.073	1.791	0.037
EBD→OP	−0.045	0.024	1.838	0.033
IN→OP	0.229	0.120	1.920	0.027
Size→OP	0.184	0.120	1.531	0.063

### Discussion and Implications

The novelty of the current study lies in the investigation of the association between online business capabilities, technological opportunism, and organizational performance. As per the authors’ knowledge, the current study is the first-ever testing these relationships in the context of United Arab Emirates, thus contributing to a better understanding of the instrumental nature of online business processes and willingness for technological development toward boosting organizational performance in the manufacturing sector. The study found that online procurement capability was highly significant in boosting organizational performance. This suggests that manufacturing businesses deploying online procurement processes to communicate procurement matters with suppliers, sharing production schedules and order catalogs with suppliers for material management, and exchanging information on material for demand management were able to bring noticeable improvement in their organizational performance. Compared to other factors investigated in the current study, online procurement capability was more significant in boosting organizational performance (*β* = 0.972; *t* = 13.792), thus highlighting the strategic significance of digitization of procurement activities. The finding strengthens prior studies that empirically indicated the vitality of online procurement processes in furthering end performance prospects (Chang and Wong, 2010; Quesada et al., 2010; Sánchez-Rodríguez et al., 2019; Madzimure et al., 2020). Accordingly, the strong association between online channel management capability and organizational performance (*β* = 0.317, *t* = 5.144) suggests that online transaction processing, sharing of marketing policies with distributors electronically, online exchange of pricing and product launch and completion details, and providing production schedules to distributors online to support order fulfillment can boost the overall performance of manufacturing organizations. The result strengthens prior literature (Oh et al., 2012; Zhu et al., 2020), suggesting that online channel management can strengthen internal processes to improve engagement with the distributors which contribute to augmenting organizational performance.

Similarly, online service delivery capability and organizational performance nexus also were found to be significant (*β* = 0.159, *t* = 3.919). The results are parallel to prior investigations confirming the prominence of online service delivery for organizational benefits (Attaran and Woods, 2019; Martín-Peña et al., 2019; Suhartanto et al., 2019; Ho et al., 2020). The findings suggest that the digital business capability to perform online services pertaining to customer communication, demand tracking, and after-sales support can help enhance organizational performance. The study showed that manufacturing businesses with online communication facilities for customer interaction and the provision of value-added services for customer feedback and after-sales services enabled them to maximize their end results. Importantly, to a larger extent, technological opportunism also emerged as a prominent factor in boosting organizational performance. The direct association between technological opportunism and organizational performance underlines that businesses actively on the lookout for appropriate technologies and developing their systems were able to harness their end performance. Parallel to this, manufacturing enterprises that were high in technological opportunism could better capitalize on the use of online channel management and online service delivery capabilities to boost end performance. Thus, an active outlook toward technological developments and their appropriate application can help businesses strengthen the capitalization of online channel management and service delivery options to better contribute to organizational performance. However, contrary to our speculation, technological opportunism did not moderate the online procurement capability and organizational performance association. A plausible reason for this could be the limited consideration of sampled organizations on the equal significance of technological opportunism in this regard.

### Theoretical Implications

Scarcity of empirical work is evident to understand the instrumental nature of e-business processes toward improving organizational performance. To offer some important theoretical contributions, the current study has attended to this rarely investigated domain (Devaraj et al., 2007; Zhu et al., 2020). At first, the present study provides a robust theoretical framework based on the assertions of resource orchestration theory regarding e-business processes. The study both strengthens and extends the application of this theory in the IS literature by explaining how e-business capabilities can be considered important resources to further organizational performance. Accordingly, the current study investigated the three individual e-business processes as notable capabilities to provide a better scholarly understanding of their importance, respectively, rather than considering them as a single process (Saeed et al., 2005; Šaković Jovanović et al., 2020). Similarly, while extant literature could only educate us on the importance of e-business entity as a whole (e.g., Rai and Tang, 2010), the current study offers a deeper understanding of how online capabilities related to suppliers, distributors, and customers can help leverage organizational performance. Henceforth, the study provides novel insights into the role of electronic inter-firm processes to offer an efficient and effective interface for enhancing business value and performance.

The current study has underlined the significance of organizations with a proactive lookout for technological developments and their capitalization. Furthermore, the study has also addressed the paucity of empirical attention toward technological opportunism (Lucia-Palacios et al., 2014), both in terms of its direct as well as moderating effect. To the best of our knowledge, the current study is the first to investigate the moderation of technological opportunism on the nexus between online procurement capability, online channel management, online service delivery, and organizational performance. The findings outline how leveraging these e-business processes can help bring promising results for organizations, particularly those with an eagle-eye for identifying and capitalizing on appropriate technological developments.

### Practical Implications

The study’s findings offer some key insights for managerial decision making in production businesses to boost organizational performance. At first, the current study provides a framework for managers to design and develop the framework for e-business processes that would help them create a mechanism to bring effectiveness and efficiency in the business operations, thus boosting performance. For instance, the development of online capabilities that enables a business to perform product realization, service identification, transaction processing, product coordination, material management, and demand planning can be critical for strengthening procurement operations, thus boosting organizational performance. Secondly, the study’s findings imply that operations managers should develop online channel management applications that could assist in matters related to unified promotion, product launches, pricing, and online transaction and order fulfillment entities. This holds a particularized importance as it will enable the integration of relational resources with the technical to boost digitization in the organization, thus improving business functioning. This will also help businesses to actively share information with the distributors and other transactional activities, aiding in reducing operational costs.

Thirdly, the findings of the study imply the need for furthering online service delivery capabilities for effective customer communication, sales, after-sales, and demand tracking. Such an online prospect would help production-based businesses in particular to timely respond to customer queries and ensure that they are well connected with the organization. These e-business processes would provide a detailed blueprint for managers to understand and effectively manage procurement, channel management, and service delivery prospects for operational excellence, thus improving organizational performance. Lastly, the study’s findings also imply that organizations not only integrate technology in the business processes but also develop a process that facilitates the prompt identification and capitalization of relevant technological developments. Managers may work to develop a sense of understanding and willingness among the top management toward the importance of implementing technological advancements in the business for competitive results and high performance. Training programs may be considered to educate and harness technological opportunism across the organization to ensure maximum utilization of e-business processes for organizational performance.

## Limitations and Scope for Future Studies

The findings of the study are significant and equally crucial for related researchers and organizational scholars. However, some important limitations must be acknowledged. First, although the investigated variables of the study can be termed adequate to investigate the organizational performance of businesses engaged in utilizing technological capabilities for procurement, channel management, and service delivery, it is believed that other variables can also contribute to the framework. Accordingly, the study tested the framework across United Arab Emirates, and therefore, future studies may find it interesting to test it across different demographics to enhance the generalizability of the findings. Therein, scholars may also consider conducting a cross-country investigation of the framework. Furthermore, the data were collected at a single point, and therefore, future studies may consider a time-lapse investigation. Lastly, contrary to our speculation, technological opportunism did not moderate the online procurement capability and organizational performance association. Future studies are encouraged to investigate the framework further to confirm the interacting effect of technological opportunism.

## Data Availability Statement

The original contributions presented in the study are included in the article/supplementary material; further inquiries can be directed to the corresponding author.

## Author Contributions

MA-O: literature review. MA: hypothesis development. UA: English and discussion apart. MP: data analysis. QA: references and data collection. All authors contributed to the article and approved the submitted version.

## Conflict of Interest

The authors declare that the research was conducted in the absence of any commercial or financial relationships that could be construed as a potential conflict of interest.

## Publisher’s Note

All claims expressed in this article are solely those of the authors and do not necessarily represent those of their affiliated organizations, or those of the publisher, the editors and the reviewers. Any product that may be evaluated in this article, or claim that may be made by its manufacturer, is not guaranteed or endorsed by the publisher.
